# Partial Resection of the Urinary Bladder

**Published:** 1882-07

**Authors:** 


### PARTIAL RESECTION OF THE URINARY BLADDER.

This was the subject for discussion in the Society of Physicians at Budapest, January 7th, introduced by Dr. Fischer. (*Pest. Med. Chirurg. Presse.*)

This operation was first performed by Pott, afterward by Chopart and Desault. At present, however, it is seldom undertaken. Dr. Fischer has experimented on eight dogs, and comes to the following conclusions:

The excision of a portion of the bladder is not so dangerous as the opening accompanying the high operation for cystotomy; the repair occurs immediately, when the edges of the wound have been accurately adjusted. In uniting the edges the interrupted suture was found to answer as well as the continuous.

The author proposes the following indications for resection of the bladder:

1. Traumatic lesions, with crushing of the edges of the wound.
2. A diverticulum of the bladder, in case of encapsulated stone.
3. The protruding lips of vesical hernia.
4. Extensive general dilatation of the bladder when the cause is a remote one.
5. Neoplasms situated deep in the texture of the bladder.
6. Various vesical fistulæ.
7. Destructive ulcer of the bladder, threatening rupture.

The difficulty necessarily attending the diagnosis of part of these indications is much against them.—*Cincinnati Lancet and Clinic.*

